# One-year outcome of frailty indicators and activities of daily living following the randomised controlled trial; *“Continuum of care for frail older people”*

**DOI:** 10.1186/1471-2318-13-76

**Published:** 2013-07-22

**Authors:** Kajsa Eklund, Katarina Wilhelmson, Helena Gustafsson, Sten Landahl, Synneve Dahlin-Ivanoff

**Affiliations:** 1 Vårdalinstitutet, The Swedish Institute for Health Sciences, University of Gothenburg and Lund, Lund, Sweden; 2 Department of Clinical Neuroscience and Rehabilitation, The Sahlgrenska Academy at University of Gothenburg, Gothenburg, Sweden; 3 Department of Public Health and Community Medicine/Social Medicine, The Sahlgrenska Academy at University of Gothenburg, Gothenburg, Sweden; 4 Department of Physiology, The Sahlgrenska Academy at University of Gothenburg, Gothenburg, Sweden; 5 Department of Medicine, Sahlgrenska University Hospital/Mölndal, Mölndal, Sweden

**Keywords:** Integrated care, Health care chain, Rehabilitation, Independence, Aging in place, Frail older people

## Abstract

**Background:**

The intervention; “*Continuum of Care for Frail Older People*”, was designed to create an integrated continuum of care from the hospital emergency department through the hospital and back to the older person’s own home. The aim of this study is to evaluate the effects of the intervention on functional ability in terms of activities of daily living (ADL).

**Methods:**

The study is a non-blinded controlled trial with participants randomised to either the intervention group or a control group with follow-ups at three-, six- and 12 months. The intervention involved collaboration between a nurse with geriatric competence at the emergency department, the hospital wards and a multi-professional team for care and rehabilitation of the older people in the municipality with a case manager as the hub. Older people who sought care at the emergency department at Sahlgrenska University Hospital/Mölndal and who were discharged to their own homes in the municipality of Mölndal, Sweden were asked to participate. Inclusion criteria were age 80 and older *or* 65 to 79 with at least one chronic disease and dependent in at least one ADL. Analyses were made on the basis of the intention-to-treat principle. Outcome measures were ADL independence and eight frailty indicators. These were analysed, using Chi-square and odds ratio (OR).

**Results:**

A total of 161 participated in the study, 76 persons allocated to the control group and 85 to the intervention group were analysed throughout the study. There were no significant differences between the groups with regards to change in frailty compared to baseline at any follow-up. At both the three- and twelve-month follow-ups the intervention group had doubled their odds for improved ADL independence compared to the control (OR 2.37, 95% CI; 1.20 – 4.68) and (2.04, 95% CI; 1.03 – 4.06) respectively. At six months the intervention group had halved their odds for decreased ADL independence (OR 0.52, 95% CI; 0.27 – 0.98) compared to the control group.

**Conclusions:**

The intervention has the potential to reduce dependency in ADLs, a valuable benefit both for the individual and for society.

**Trial registration:**

ClinicalTrials.gov: NCT01260493

## Background

The current trend in Western societies facing a growing proportion of older people is to support older people to remain in their homes as long as possible, so-called ageing in place [1]. Research has confirmed that the home is a central and meaningful place for older persons [2]. It is a place for activities, where events occur on the old peoples’ own terms and where they feel secure [2]. Haak et al. [2] found that independence in daily activities among the very old (80+) is strongly linked to aging in place, and that independence is highly valued and reinforces the older person’s sense of self. It is therefore essential to enable them to continue performing daily activities in their own homes even when they become frail.

Older people comprise a group whose reserve of strength is decreasing, and whose activity and participation levels will deteriorate with increasing frailty [3,4]. Frail older people are at high risk of developing chronic diseases, multi-morbidity and functional impairments. This often leads to dependence in daily activities [5-7]. The frail older person needs care from many different caregivers at different care levels and with different competences, such as gerontology, geriatrics, internal medicine, rehabilitation, nursing and social care services. It is important that this care is integrated in order to reduce fragmentation and to improve continuity and coordination of care [8].

In Sweden, “health care chains” have become an important way of integrating health care [9]. A health care chain is defined as “coordinated activities in the health care systems linked together to achieve a final result of good quality for the patient” [9]. A well-functioning chain implies that the care is seen as a continuum between different caregivers and care levels, and it is assumed that one caregiver of high quality is not enough to ensure good care. One way of enhancing continuity and integration is to use geriatric screening and multidimensional assessment, a method which involves different categories of caregivers and improves communication [10]. One important component in integrated care programmes is case management (CM), defined as the coordination of various system components for a successful outcome [11]. A review of interventions to maintain independent living among frail community-dwelling older persons points out promising features of interventions which include multidisciplinary and multi-factorial, individualized assessment and intervention, case management and long-term follow up [12].

The evidence-based findings from the aforementioned studies guided us in the design of the randomised, two-armed intervention study of frail community-dwelling older people: “*Continuum of Care for Frail Older People*” [13], creating a continuum of care from the hospital emergency department to the older person’s own home. One hypothesis of the project was that an intervention programme for frail older people can maintain functional ability. The present study aims at evaluating the effects of the intervention on functional ability in terms of activities of daily living and frailty up to one year later.

## Methods

### Study design

The study is a randomised non-blinded controlled trial with participants randomised to two study arms, one intervention group and one control group with follow-ups at three-, six- and 12 months. Main reason for non-blinding were that the participant revealed the allocated group assignment at follow-ups but also, we assumed there would be less attrition if the older person could meet the same research assistant at most of the follow-ups. The study was conducted during the period October 2008 to November 2011. The Regional Ethical Review Board in Gothenburg, ref.nr. 413–08, approved the study, and written informed consent was obtained from the participants.

### Participants and setting

The study group includes older people who sought care at the emergency department at Sahlgrenska University Hospital/Mölndal and who were discharged to their own homes in the municipality of Mölndal, Sweden. Inclusion criteria were age 80 and older *or* 65 to 79, with at least one chronic disease and dependent in at least one activity of daily living. Exclusion criteria were acute severe illness with immediate need of assessment and treatment by a physician (within ten minutes), dementia (or severe cognitive impairment, clinically assessed by the nurse with geriatric competence at the emergency department), and palliative care. To get data about the older population attending the ED, all persons 65 years and older were registered over a month proceeding the start of the study. During January 2007, 0.5% were in need of assessment and treatment by a physician within ten minutes and a small fraction came in outside of daytime hours. The study group comprised a representative sample of frail older people at a high risk of future health care consumption [13].

### Intervention group

The intervention involved collaboration between a nurse with geriatric competence at the emergency department, the hospital wards and a multi-professional team for care and rehabilitation of the older people in the municipality with a case manager as the hub. Together a continuum of care was created for the older person from the emergency department, through the hospital ward and on to their own homes. The intervention had a person-centred approach [14] with shared decision-making throughout the care chain. The multi-professional team included professionals in nursing (the case manager), occupational therapy, physiotherapy and social work, for intervention components see Table 1.

**Table 1:** Components of ordinary care and Continuum of care

**Intervention components**	**Ordinary care/control group**	**Continuum of care for frail older people**
Frailty screening and geriatric assessment at emergency department (ED) by nurse with geriatric competence	No	Yes, need of rehabilitation, nursing, geriatric and social care
Case manager (CM) in the municipality with multi-professional team for care and rehabilitation	No	Yes
Hospital care if needed and rehabilitation at hospital if needed	Yes	Yes
Track 1. In need of hospital care: information transfer to ward and case manager in the municipality. CM responsible for contacting the ward and the patient in order to prepare the municipality in good time before being discharged	No	Yes
Track 2. Not in need of hospital care: information transfer to case manager in the municipality		
Care planning	Yes, at hospital before discharge if assessed as having new or changed needs of home care by a team from the municipality consisting of different professionals (nurse, occupational therapist, physiotherapist or social worker) responsible for all care planning at the hospital Not for persons with no need of hospital care.	Yes, at home for both tracks within a couple of days of discharge, based on ED frailty screening and a comprehensive geriatric assessment by CM and team
Rehabilitation in the municipality if assessed as needed at care planning	Yes	Yes
Follow-ups other than research	Yes, after rehabilitation	Yes, by CM within a week after care planning and then at least every month for a year

### Control group

The control group received ordinary care, for components see Table 1.

### Procedure

The participants were recruited whilst in the emergency department by nurses with geriatric competence during the daytime on weekdays. Patients attending the emergency ward at other hours were recruited by either a visit to the wards or by letter if discharged before recruitment (n = 17). The nurse informed the participants about the study both verbally and in writing. The information included a description of the study, how it would be conducted and what was expected of persons who agreed to participate. There were opportunities to ask questions if anything was unclear. It was stressed, both in the verbal and the written information, that participation was voluntary. The persons who accepted to participate in the study were randomised to either the intervention or the control group by the nurse, by using sealed opaque envelopes. All participants signed a written consent form.

### Data collection and outcome measures

The baseline data for the intervention group were collected by the multi-professional team as part of their comprehensive geriatric assessment. The baseline data for the control group and all follow-ups for both groups were collected by research assistants, who were either registered occupational therapists or nurses. Baseline data (= interviews and assessments) were predominantly collected within a week following discharge, but in three cases data collection was postponed one to two weeks in view of the strain of the participants. Follow-up data was collected three-, six- and 12 months following discharge.

All data were collected in the participants' homes and all interviewers were well trained in interviewing, assessing and observing, according to the guidelines for the different outcome measurements. To ensure as much standardization of the assessments as possible, study protocol meetings were held regularly throughout the study.

#### Activities of Daily Living (ADL)

The degree of independence was measured as a sum of activities managed independently using the ADL staircase [15]. The ADL staircase measures independence of, or dependence on, another person in five personal ADL items (i.e. bathing, dressing, going to the toilet, transferring, and feeding) Katz et al. [16], extended with four instrumental items (i.e. cleaning, shopping, transportation, cooking). Dependence was defined as a state in which another person was involved in the activity by giving personal or directive assistance. The ADL staircase is administrated using a combination of interview and observation. The validity and reliability of the ADL staircase is good for the age group in present study [17,18] and has recently been found to predict mortality [19]. The number of activities managed independently (0–9) was summarized at baseline and at each follow-up.

#### Frailty

Frailty was measured as a sum of eight core frailty indicators: weakness, fatigue, weight loss, low physical activity, poor balance, low gait speed, visual impairment and cognitive impairment. Cutoffs for weakness, such as a grip strength of less than 13 kg for women and 21 kg for men for the dominant hand and 10 kg for women and 18 kg for men for the non-dominant hand, were measured using a hand dynamometer [20], Fatigue was noted if a participant answered yes to the question: “Have you suffered any general fatigue or tiredness over the last three months?” [21], Weight loss was noted if a participant answered yes to the question: “Have you suffered from any weight loss over the last three months? [21]. Low physical activity was defined as one to two walks per week or less. Poor balance involved a score of 47 or lower on the Berg balance scale [22]. Low gait speed was walking four meters in 6.7 seconds or slower [23]. Visual acuity was measured with the KM chart, and visual impairment was a visual acuity of ≤0.5 in both eyes [24,25]. Cognitive impairment was defined as < 25 points in the Mini Mental State Examination [26]. For more details, see the study protocol [13]. The sum of frailty indicators was the total number of indicators exceeding the cut off for frailty (0–8), summarized at baseline and at each follow-up. Level of frailty was operationalised as; non-frail = 0 frailty indicator, pre-frail = 1–2 indicators, frail = >2 indicators.

### Sample size

A power calculation was made based on the Berg balance scale (one of the frailty indicators, range 0–56), with an assumed mean of 32 for the intervention group and 28 for the control group (15% difference), and a standard deviation of 8 in both groups. To be able to detect a difference between the intervention and control groups with a two-sided test and with a significance level of alpha = 0.05 and 80% power, at least 65 people were needed in each group. For more details, see the study protocol [13].

### Statistical analyses

The analyses were made on the basis of the intention-to-treat principle. The basic assumption for imputing data was that older adults are expected to deteriorate over time in the natural course of the ageing process. Therefore, the imputation method chosen was to replace missing values in the sum of ADL activities managed independently and the sum of frailty indicators with a value based on the median change of deterioration (MCD) between two measuring points (baseline to follow-up and between two follow-ups) of all who participated at both measuring points. The MCD was added to the last actual individual value recorded, and imputed, substituting missing data at the three-, six- and 12-month follow-ups. An exception was made for missing values due to death, which were imputed with the worst case rank at each follow-up. Sensitivity analyses were made comparing MCD analysis with complete case analysis and showed aligned trends [27]. This, in combination with our stated basic assumption and that MCD is a more conservative form of worst case deterioration [28] guided the final preference of the MCD method used in the analyses presented. In three cases baseline data were missing, but the participants did have follow-up data. In these cases values for baseline were retrieved from medical records.

The baseline and dropout characteristics of the two groups were compared using Chi-square or Fischer’s exact test. The number of participants that had improved maintained or decreased their degree of ADL independence, and the number that had improved, maintained or decreased their level of frailty compared to baseline was calculated during the course of the study. The outcomes were analysed using Chi-square and odds ratio (OR) to compare between groups. Two-sided significance tests were used throughout. A p-value ≤ .05 was considered statistically significant, and a 95% confidence interval (CI) is provided. In addition, ordinal regression analyses were made to test for confounders. Statistical analyses were made using PASW Statistics, version 18.0 (SPSS Inc., Chicago, IL).

## Results

**Figure 1:**
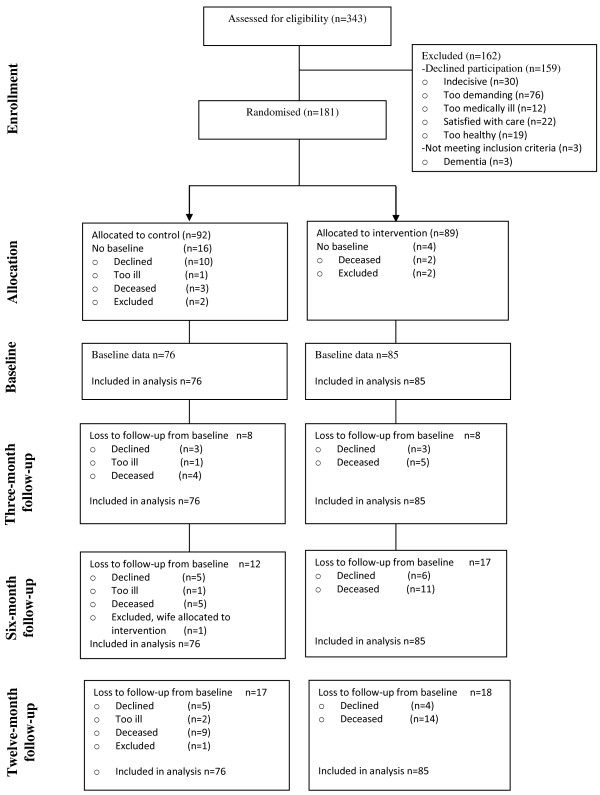
Flow-chart of randomization, allocation, follow-ups and analysis for the study period.

During the inclusion period 1 445 older persons living in the municipality sought care at the emergency department. Of these, 343 persons met the inclusion criteria and were asked for participation, 181 persons consented to participate, 159 declined, and three were found to have dementia when further assessed. The flow of participants through the study is shown in the CONSORT diagram, Figure 1. At the time of the baseline assessment, twenty of the participants were not assessed; for reasons, see Figure 1. Thus, in all 161 participated in the study, of which 76 persons were allocated to the control group and 85 to the intervention group and were analysed throughout the study. The median age of the non-participants was somewhat lower than that of the participants, but the age range was similar. The most common reasons for not participating were that the study seemed too demanding. For other reasons see Figure 1. All participants assigned to intervention participated in it. All participants in the intervention group had care planning at home, 50% (n = 38) among the control group had care planning at hospital. There were no significant differences in baseline characteristics between the participants in the two study groups, see Table 2.

**Table 2:** Baseline characteristics of study participants, their proportions and p-value for differences between groups

**Characteristics**	**Control group n = 76**	**Intervention n = 85**	**p-value**
Female, %	55	55	0.997
Living alone, %	60	60	0.946
Academic education, %	16	12	0.458
Self-rated health, good, %	29	39	0.187
Non-frail, %	0	5	0.055
Pre-frail, %	24	26	0.747
Frail, %	76	69	0.326
Visual impairment, %	81	70	0.753
MMSE, ≤25, %	7	16	0.080
ADL, independent in all activities, %	26	20	0.342
Discharged home directly from ED, %	15	16	0.584
Length of hospital stay, median	4	5	0,267

*MMSE* Mini Mental State Examination, *ADL* Activities of Daily Living.

There was no significant difference in drop-out rates between the control and intervention groups at the three-month, six-month and twelve-month follow-ups. The drop-outs at the three-, six- and twelve-months follow-ups had been significantly weaker, had poorer balance and lower gait speed at baseline compared to participants. In addition, the drop-outs at twelve months had a significantly higher proportion of weight loss at baseline. The median change of deterioration imputed for the drop-outs was one step in both the sum of ADL independence and the sum of frailty indicators between all measuring points.

There were no significant differences between the control and intervention groups with regard to improved, maintained or decreased of level of frailty compared to baseline at any follow-up, see Table 3. Most movements between levels of frailty were either from being frail to being pre-frail or vice versa. In all, three participants had moved two levels at twelve month, one from non-frail to frail (intervention group) and two from frail to non frail (control group).

**Table 3:** Odds ratio (OR) and 95% confidence interval (CI) for changes in levels of frailty at follow-ups

**Changes in levels of frailty**	**Control n= 76**			**Intervention n= 85**			
	**%**	**(n)**	**OR**^**1**^	**%**	**(n)**	**OR**	**(95% CI)**
**Improved**							
three-month	13	(10)	1	8	(7)	0.59	(0.21-1.64)
six-month	17	(13)	1	12	(10)	0.65	(0.27-1.57)
twelve-month	22	(17)	1	12	(10)	0.46	(0.20-1.09)
**Maintained**							
three-month	76	(58)	1	78	(66)	1.08	(0.52-2.25)
six-month	75	(57)	1	74	(63)	0.95	(0.47-1.94)
twelve-month	68	(52)	1	74	(63)	1.32	(0.67-2.62)
**Decreased**							
three-month	11	(8)	1	14	(12)	1.40	(0.54-3.63)
six-month	8	(6)	1	14	(12)	1.92	(0.68-5.39)
twelve-month	9	(7)	1	14	(12)	1.62	(0.60-4.32)

^1^ = reference, participants in the control group.

At both the three- and twelve-month follow-ups the intervention group had a higher OR in improved degree of ADL independence, with an OR of 2.37 (95% CI; 1.20 – 4.68) and 2.04 (95% CI; 1.03 – 4.06), respectively, see Table 4. The median improvement was one step in the control group at all follow-ups, one step at three month follow-up and two steps in the following follow-ups in the intervention group. Among those who had decreased degree of ADL independence, the intervention group had a lower OR (0.52, 95% CI; 0.27 – 0.98) than the control group at six-month. The median decrease was two steps in the control group at all follow-ups, one step at three month follow-up and two steps in the following follow-ups in the intervention group. There were no differences between the groups among those who had maintained the same degree of independence compared to baseline either at the three-, six-, or twelve-month follow-ups. During the course of the study, no participants were institutionalized.

**Table 4:** Odds ratio (OR) with 95% confidence interval (CI) for changes in degree of independence in Activities of Daily Living (ADL) at follow-ups

	**Control n=76**			**Intervention n=85**			
	**%**	**(n)**	**OR**^**1**^	**%**	**(n)**	**OR**	**(95% CI)**
**Improved ADL**							
three-month	24	(18)	1	42	(36)	2.37	(1.20-4.68)
six-month	28	(21)	1	36	(31)	1.50	(0.77-2.94)
twelve-month	24	(18)	1	39	(33)	2.04	(1.03-4.06)
**Maintained ADL**							
three-month	43	(33)	1	38	(32)	0.79	(0.42-1.48)
six-month	26	(20)	1	32	(28)	1.30	(0.66-2.59)
twelve-month	29	(22)	1	24	(20)	0.76	(0.37-1.53)
**Decreased ADL**							
three-month	33	(25)	1	20	(17)	0.51	(0.25-1.04)
six-month	46	(35)	1	31	(26)	0.52	(0.27-0.98)
twelve-month	47	(36)	1	38	(32)	0.67	(0.36-1.26)

^1^ = reference, participants in the control group.

Due to possible relevant differences at baseline between the groups, the ADL outcome was tested for confounders with MMSE, frailty and self-rated health, and the frailty outcome with MMSE and self-rated health, finding no modifying effects.

## Discussion

The evaluation of ‘*Continuum of care for frail older people’* showed that the intervention has succeeded in both improving ADL independence among its participants up to one year, and in postponing dependence in ADL up to six months. There did not appear to be any differences between the groups with regards to change in frailty as a result of the intervention.

The improvement in ADL independence is of great importance both for the persons concerned and to society. Developing a higher degree of ADL dependence according to the ADL staircase has been found to indicate increased risk of dying [19]. For the older person (80+), being independent means a lot for self-confidence and feelings of pride and satisfaction [2]. To be dependent on personal help has been found to be correlated with poor quality of life [29]. In addition, Johannesen et al. [30] found that dependence on personal help and use of home care services was primarily associated with lack of everyday life satisfaction among older persons. For the society, the improvement in ADL and the postponement of increased dependence should lead to less use of the municipal home care service and thereby a less stress on economic resources of the same [31]. Further, relatives often carry a heavy burden as informal caregivers to a wife, husband or a parent who is dependent on help in ADL [32]. The improvements in ADL independence following the intervention could lessen this burden among caregivers if the intervention is implemented in ‘real life’, that is, implemented on a regular basis. Summarizing thus far, due to the foreseen increasing proportion of older persons in Sweden, as in the rest of the western world, these findings of ADL improvement and postponement of dependency show that the ‘*Continuum of care for frail older people’* has the means to contribute beneficially both to the individual and society at large when implemented in ‘real life’.

Though no differences between the intervention and control as far as frailty were found, both groups moved between frailty levels during the course of the study. Gill et al. have earlier shown that frailty is a dynamic process characterized by frequent transitions between frailty levels [33] and that recovery from frail and pre-frail stages is diminished by intervening hospitalizations [34]. Even though to a small extent, participants in both groups in present study improved regarding their frailty. When looking at the results, one has to keep in mind that the control group received ordinary care, which of course should also be beneficial. Ordinary care for frail older persons in Sweden does in fact contain some of the same components as in the continuum of care, that is, rehabilitation at different care levels, care planning and municipal home care for those in need, all important for meeting their needs. However, it does not include frailty screening and geriatric assessment at the emergency department, a case manager who integrates the care to monitor that the frail older persons get the right care at the right time, care planning with geriatric assessment at home and to be followed up. A three-armed study with a group receiving neither ordinary care nor the integrated care chain would have given us an estimate of the effect of the ‘*Continuum of care for frail older people’*, but this would of course not have been ethical. The good quality of ordinary care in Sweden could be another reason why there were no differences in frailty between the control group and intervention group at any of the follow-ups [35]. Also, the majority of the participants were already frail and dependent in ADL at baseline. For such a frail group, the risk of adverse events, such as acute illness and increased frailty, is recognized [36]. Thus, even with an intervention with well-integrated care, the effects on frailty might be small or non-existent. It is known that interventions are in fact more efficient when targeting older people who are at the beginning of developing frailty, i.e. the pre-frail [37]. Yet another possible explanation for the non-difference in frailty could be that the outcome measure was not sensitive enough. The power calculation was based on one of the frailty indicators, not the sum of eight. Also, by focusing on physical frailty as proposed by Fried [36] and not a more multi dimensional definition of frailty, as proposed by Gobbens [38] effects can have been overlooked.

The positive outcome compared to ordinary care is most likely explained by the integration of care, as solitary components in complex interventions are difficult to single out as successful factors. Still, likely contributing factors could be the early identification of needs by the frailty scanning and geriatric assessment already at the emergency department, patient planning based on geriatric assessment in the older person’s home and regular follow-ups. In a meta-analysis of complex interventions targeting older people [37], it was found that the overall impact of such interventions on physical function, e.g. ADL, was beneficial. However, when different contexts were analysed separately, the impact on ADL varied. When the geriatric assessment focused on older adults selected as frail or when the interventions only included community care after hospital discharge, no impact on ADL was seen [37]. Thus we argue that it is important that the whole integrated care chain with all parts of the continuum of care is implemented as intended. The fidelity of the implementation of the whole care chain of ‘*Continuum of care for frail older people’* has been studied alongside the RCT study [39]. The results show that fidelity was generally high. A review of other integrated care programmes for frail older persons, evaluated by randomised controlled studies, showed that five out of eight studies indicated positive effects for the older person and no negative effects. Positive effects were reported in relation to medication, client satisfaction, ADL, quality of life and depression [40]. These findings coincide with results from the *‘Continuum of care for frail older people’* study both in terms of ADL and client satisfaction. An additional study of Berglund et al. (unpublished observation) reported that client satisfaction was significantly higher in the intervention group than in the control group.

A weakness of the present study is that it was not possible to keep the interviewer blinded to group assignment during the follow-ups, thus risking biased results. The participants revealed the allocated group assignment at follow-ups and we assumed there would be less attrition if the older person could meet the same research assistant at most of the follow-ups. The second reason seems to have been a correct assumption since the drop-out rate was fairly low considering the age and health status of the participants; 22% in the control group and 21% in the intervention or 9% and 4% respectively when those who died are omitted. This is, in turn, an important quality factor when looking at the strengths and weaknesses of a study. To minimize the risk of bias due to non-blinding, valid and reliable questionnaires and measurements were used and study protocol meetings were held throughout the study period to ensure standardization of the assessments. In addition, the research assistants had university degrees and were well trained in assessing frail older persons. All in all, the non-blinding of the research assistants is a limitation to the study, but we believe that the monitoring of their assessments modifies this limitation to some extent.

Another limitation in present study is that the ADL staircase has fewer I-ADL items than other internationally used ADL instruments have. When performing research with frail older people it is a challenge to balance between that they are easily exhausted and a need of detailed information. The four items in the ADL staircase correspond to the same items in both Lawton [41] and Level of Rehabilitation Scale [42], but does not include other important items such as making phone calls or doing laundry. When deciding to use the ADL-staircase this difference was considered but fewer items and its good validity in the age group in present study was considered to surpass its limitations.

Even if the drop-out level was low, as stated above, we believe missing data had to be imputed. The choice of imputation was based on the assumption that older persons are expected to deteriorate over time. The analysis of the drop-out subjects at the three-, six- and twelve month follow-ups reflects this by showing that these persons had significantly worse baseline measures than those participants who completed the study. Also, we know that the main reason for drop-out was because the person had deceased. Therefore, we assumed that missing data was not a random occurrence [43]. The drop-out subjects in intervention studies targeting older persons are more likely to have worse outcomes which has been confirmed earlier [44]. This selection bias further justifies our chosen imputation method, MCD. Other imputation methods such as the EM-algorithm, would also have been appropriate, since it would have had estimates with more refined imputation values [45] compared to the more crude MCD method. Even though the MCD method clearly has a limitation of being crude we believe that it is useful in our study since about half of the drop-out subjects had died and subsequently not at random. We believe that by giving them the worst rank and the drop-out the MCD method gives a rather fair estimate of missing values. In addition, the sensitivity analysis showed aligned trends when comparing with complete cases.

The intervention has been implementing in full scale in Mölndal, performing an implementation study alongside. Reasons why independence increased among the intervention participants at both three and twelve month follow-ups, but not at six months will be investigated. At first glance this outcome pattern seems inconsistent; but the difference in ADL performance between the groups does remain at six months even though it is by preventing dependence. As of now, we can only speculate about this pattern. But a plausible explanation is that the care planning at home identified relevant ADL problems that the older adult was motivated in starting to use an assistive device to overcome the problem. Earlier research has shown that assistive devices initially are well used but then comes a period of frustration and not using them consistently before getting used to them on a day to day basis. This process is individual but can take up to a year [46]. This too will be followed in the implementation study.

The municipality of Mölndal comprises both urban and rural areas. As the prevalence of dependence in ADL among older people in Sweden is higher in rural areas [47], Mölndal could be seen as a typical area of ADL performance in Sweden, since it includes areas with both a lower and a higher prevalence of dependence in ADL. Among non-participants, reasons for declining participation in the study were both that health was too bad and too good. Thus, non-participants were both in worse health and healthier than the participants [13]. It could be argued that by excluding ED visitors with severe illness the sample was unique, but preceding the study it was estimated that only 0.5% were in need of physician within 10 minutes. Therefore, it can be assumed that the participants can be seen as a fairly representative sample of the frail older population when taking into account that persons with dementia and palliative care were excluded on ethical grounds.

## Conclusions

The intervention *‘Continuum of care for frail older people’* showed that the integrated intervention improved independence in ADL up to twelve months and postponed dependence up to six months. Thus, the intervention has the means to support the frail older to age in place; a valuable benefit both to the individuals concerned and for society at large.

## Competing interests

The authors declare that they have no competing interests.

## Authors’ contributions

KE drafted the manuscript. KW was project leader of the implementation. KE, KW, HG and SL participated in the research design and implementation of the study. KE, KW and SDI performed the statistical analysis. SDI was the main research designer and led the implementation of the study. All authors contributed to the writing and review of the manuscript. All authors read and approved the final manuscript.

## Pre-publication history

The pre-publication history for this paper can be accessed here:

http://www.biomedcentral.com/1471-2318/13/76/prepub
